# CD161 expression in tuberculosis and functional effects of CD161–LLT1 blockade

**DOI:** 10.3389/fcimb.2026.1962964

**Published:** 2026-10-28

**Authors:** Yuhan Wang, Yayan Niu, Jinjuan Liu, Yanjun Feng, Yan Sun, Mingwei Du, Shengli Gao, Xueguang Zhang, Lei Cao, Meiying Wu

**Affiliations:** 1 Department of Tuberculosis, The Fifth People’s Hospital of Suzhou/The Affiliated Infectious Diseases Hospital, Suzhou Medical College of Soochow University, Suzhou, China; 2 Department of Tuberculosis, Suzhou Medical College of Soochow University, Suzhou, China; 3 The Ninth Affiliated Hospital of Soochow University, Suzhou, China; 4 Jiangsu Institute of Clinical Immunology & Jiangsu Key Laboratory of Clinical Immunology, The First Affiliated Hospital of Soochow University, Suzhou, China; 5 Suzhou Key Laboratory for Tumor Immunology of Digestive Tract, The First Affiliated Hospital of Soochow University, Suzhou, China

**Keywords:** CD161, immune checkpoint, Klrb1, LLT1, monoclonal antibody, soluble CD161, tuberculosis

## Abstract

**Introduction:**

CD161 (KLRB1) is a C-type lectin-like immune receptor expressed on multiple immune-cell subsets and has context-dependent immunoregulatory functions.

**Methods:**

We generated human anti-CD161 monoclonal antibodies, established a customized in-house sandwich ELISA for soluble CD161 (sCD161), and identified antibodies that block the CD161–LLT1 interaction. In a single-center cross-sectional cohort, we evaluated 63 untreated patients with active pulmonary tuberculosis (TB), together with 52 healthy controls (HCs), 6 patients with community-acquired pneumonia (CAP), and 5 individuals with latent TB infection (LTBI) for plasma sCD161. Membrane CD161-positive cell frequencies were assessed in 57 TB patients and 27 HCs. CD161 distribution in lung and lymph-node tissues and the effects of CD161–LLT1 blockade on cytokine responses in an ESAT6-CFP10 antigen-stimulated PBMC system were also examined.

**Results:**

Plasma sCD161 levels were higher in TB patients, particularly those classified as severe by radiographic criteria, and showed weak inverse associations with selected membrane CD161-positive T-cell frequencies. CD161 staining was increased in perigranulomatous regions and decreased in necrotic regions. Blocking CD161–LLT1 altered cytokine secretion, with reduced IFN-γ, IL-6, and TNF and increased IL-17A in selected antibody conditions.

**Discussion:**

These findings indicate that circulating sCD161 is associated with TB and radiographic disease severity in this cohort, while CD161–LLT1 blockade modulates cytokine responses in an antigen-stimulated PBMC system. Larger, independently validated and longitudinal studies are required to determine the diagnostic and treatment-monitoring utility of sCD161 and to define the cellular mechanisms underlying CD161–LLT1 signaling in TB.

## Introduction

Tuberculosis (TB), caused by Mycobacterium tuberculosis (Mtb), is a chronic infectious disease that remains a major global public health threat. Mtb is an intracellular pathogen capable of inducing granuloma formation, organized aggregates of macrophages, neutrophils, and lymphocytes. Mature granulomas can contain infection when adaptive immunity is effective, whereas insufficient immune control can disrupt granuloma structure and contribute to active or disseminated TB (Lyu J. et al., 2024). Thus, TB pathogenesis reflects a dynamic interaction between Mtb and host innate and adaptive immune responses, particularly T-cell-mediated immunity.

T cells are classified into αβ and γδ subsets according to their T-cell receptor (TCR) composition, and both contribute to TB immunity. αβ T cells recognize peptide antigens presented by major histocompatibility complex (MHC) molecules and mediate antigen-specific adaptive responses, whereas γδ T cells recognize diverse non-peptide antigens, including phosphoantigens and lipid-associated molecules, and can provide rapid effector functions at the interface of innate and adaptive immunity. CD4^+^ T cells promote antimycobacterial immunity largely through cytokines such as IFN-γ and TNF-α that activate infected macrophages and support intracellular bacterial control. CD8^+^ T cells recognize MHC class I-presented antigens and contribute to host defense through perforin/granzyme-mediated cytotoxicity, production of IFN-γ and TNF-α, and other effector mechanisms directed against infected cells and intracellular Mtb (Lefrançais et al., 2025). CD8^+^CD161^+^ T cells comprise functionally heterogeneous populations with cytotoxic, memory, and tissue-homing characteristics, and their contribution to antimicrobial immunity may depend on the local activation state (Smith et al., 2010; Konduri et al., 2020; Lyu M. et al., 2024). γδ T cells can rapidly release cytotoxic molecules and produce IFN-γ, TNF-α, and IL-17, whereas NK cells contribute to early infection control through cytotoxicity and IFN-γ secretion (Ruibal et al., 2021).

Current diagnosis of active TB is multimodal and integrates clinical presentation, radiological findings, and microbiological testing. Symptoms such as persistent cough, fever, night sweats, fatigue, and weight loss can raise clinical suspicion, while chest radiography and computed tomography (CT) provide complementary evidence of pulmonary disease. Microbiological approaches include smear microscopy and culture for Mtb, together with rapid molecular tests based on nucleic-acid amplification (NAAT), while phenotypic and genotypic methods can be used to determine drug resistance. Recent diagnostic strategies also emphasize sputum-sparing or non-sputum molecular testing and host-based biomarkers, particularly for paucibacillary disease and patients from whom adequate respiratory specimens are difficult to obtain. In contrast, immune-based tests such as the tuberculin skin test and interferon-γ release assays primarily indicate Mtb infection and do not, by themselves, establish active TB (Kontsevaya et al., 2024; WHO Guidelines Approved by the Guidelines Review Committee, 2024a).

CD161 (KLRB1), also known as NKR-P1A, is a type II transmembrane C-type lectin-like receptor expressed on subsets of NK cells and T cells and has context-dependent immunoregulatory functions (Lanier et al., 1994; Wyrożemski and Qiao, 2021; Cheng et al., 2022). Engagement of CD161 by its ligand LLT1 (CLEC2D) can modulate cytotoxicity and cytokine production, but the functional outcome depends on the immune-cell subset and activation context (Aldemir et al., 2005; Rosen et al., 2005; Braud et al., 2022). CD161 expression has been associated with distinct functional states in infectious diseases, autoimmunity, and cancer. In tumors, CD161/LLT1 has emerged as an immune-regulatory axis of interest, and experimental blockade strategies have been explored mainly at the preclinical level rather than as an established clinical therapy (Basdeo et al., 2015; Mathewson et al., 2021; Braud et al., 2022; Weng et al., 2022). In chronic viral infection, perturbation of the LLT1–CD161 axis has also been linked to altered NK-cell phenotype and function, illustrating that its biological effects may vary across infectious settings (Lenart et al., 2023).

In TB, CD161 expression on peripheral blood T cells has been investigated as a potential means of distinguishing healthy individuals, LTBI, and active TB (Yang et al., 2015). CD161^+^ T cells can produce IFN-γ, TNF-α, IL-17A, and perforin and have been reported to inhibit intracellular mycobacterial growth, whereas these responses are impaired in patients with TB (Chua et al., 2012; Provine et al., 2018; Yang et al., 2021). Altered frequencies and functional states of NK and T-cell subsets have also been described during active TB (Albayrak et al., 2022). However, the peripheral and tissue distribution of CD161, the relationship between membrane-associated and circulating CD161, and the functional consequences of CD161–LLT1 blockade in TB remain incompletely defined.

We hypothesized that active TB is associated with altered membrane-associated and circulating CD161 and that circulating sCD161 is related to clinical/radiographic disease severity. We further hypothesized that interference with the CD161–LLT1 interaction would modify cytokine responses in an Mtb antigen-stimulated PBMC system. To test these hypotheses, we generated anti-human CD161 monoclonal antibodies, established a customized sandwich ELISA for sCD161, examined CD161 expression in peripheral blood and TB-associated tissues, and evaluated the effects of selected CD161-blocking antibodies on cytokine production after ESAT6-CFP10 stimulation.

## Materials and methods

### Antibodies, reagents, and cell lines

PE-conjugated anti-human CD161 (clone HP3G10), PE-Cy7–conjugated anti-human CD3 (clone SK7), FITC-conjugated anti-human CD4 (clone RPAT4), APC-conjugated anti-human CD8 (clone SK1), PerCP-5.5–conjugated anti-human δ2 (clone B6), APC-Cy7–conjugated anti-human CD64 (clone 10.1), and BV510-conjugated anti-human CD56 (clone HCD56) were purchased from BioLegend (San Diego, CA, USA). Recombinant human CD161-Fc and CD161-His proteins were obtained from Biotech Bio, Shanghai. Rabbit anti-human KLRB1 polyclonal antibody (Thermo Fisher, USA) and anti-human CD161 monoclonal antibody (clone EPR26340-6, Abcam, UK) were used. HRP-conjugated goat anti-rabbit IgG and rabbit anti-mouse IgG were purchased from Abcam. HAT and HT supplements were purchased from Sigma (St. Louis, MO, USA).

For immunohistochemistry, anti-CD3 (catalog no. ZM-0417, Zhongshan Golden Bridge, China), anti-CD8 (catalog no. 66481-1-Ig, Proteintech, Wuhan, China), and anti-CD161 (clone EPR26340-6, Abcam, UK) were used. A cytometric bead array (CBA) kit was obtained from BD Biosciences (catalog no. 560484, Franklin Lakes, NJ, USA). Recombinant Mycobacterium tuberculosis ESAT6-CFP10 fusion protein containing a His tag (Rv3875-Rv3874; catalog no. GRV75741; JenoBio Shanghai, China) was used for antigen stimulation at a concentration of 10 μg/ml. L929 fibroblast cells were stably transfected with human KLRB1 (L929/CD161 cells). All cells were maintained in RPMI-1640 (Gibco, Carlsbad, CA, USA) supplemented with 10% fetal calf serum (FCS; Zhejiang Four Seasons Green, China).

BALB/c mice (6–8 weeks old) were immunized with recombinant human CD161-Fc protein emulsified in a manganese adjuvant, followed by three booster injections at 3-week intervals. Three days after the final booster, splenocytes were fused with SP2/0 myeloma cells using polyethylene glycol (PEG). Hybridomas were cultured in HAT-DMEM medium in 96-well plates and screened by flow cytometry. Positive clones reactive to L929/CD161 cells, but not to L929/mock cells, were subcloned three times by limiting dilution. Hybridoma-derived monoclonal antibodies (mAbs) were expanded in pristane-primed BALB/c mice, and ascites-derived antibodies were purified using protein G affinity chromatography (GE Healthcare, Sweden).

### Characterization of monoclonal antibodies

Antibody binding to immobilized CD161-His protein was measured by ELISA. Western blotting and immunohistochemistry were used to assess recognition of CD161. Competitive binding was evaluated by ELISA using unlabeled mAbs and biotin-labeled mAbs. Distinct epitope recognition was inferred from competition patterns; formal epitope mapping and affinity measurements were not performed in this study.

### Establishment of a customized in-house sandwich ELISA for sCD161

Ninety-six-well plates were coated overnight with anti-CD161 mAb 14G12 (5 μg/ml). After blocking with 3% bovine serum albumin (BSA), recombinant CD161 fusion protein was incubated at 37 °C for 2 h, followed by biotin-labeled anti-CD161 mAb 5G6 (0.5 μg/ml) and HRP-streptavidin. 3,3′,5,5′-Tetramethylbenzidine (TMB) was used for color development, and absorbance was measured at 450 nm. Plasma samples were diluted 1:5 and quantified against the recombinant CD161 standard curve. The calibration range shown in **Figure 2C** was 0.0156–1 ng/ml (R^2^ = 0.9996). Intra-assay CVs for three samples were 3.0%, 3.7%, and 5.4%, and inter-assay CVs were 8.1%, 1.7%, and 9.6% (n = 10 for each sample; **Supplementary Table 2**). In the 0, 30, and 90 day stability assessment, CVs ranged from 0.7% to 8.9% across 0.5, 0.25, and 0.125 ng/ml controls (**Supplementary Table 3**). Analytical specificity was assessed against homologous soluble proteins (sCD4, sCD8, and sCD25). This assay was developed as a customized in-house research ELISA using the anti-CD161 monoclonal antibodies generated in this study.

### Study design and participants

This was a single-center, cross-sectional observational study. A total of 77 untreated patients with active pulmonary TB were recruited at the Fifth People’s Hospital of Suzhou (Affiliated Infectious Diseases Hospital, Soochow University) from May to November 2023 (**Supplementary Table 1**). TB diagnosis was based on microbiological confirmation of *Mycobacterium tuberculosis* or compatible clinical findings with characteristic chest CT abnormalities. Microbiologically confirmed pulmonary TB was defined as a positive sputum smear microscopy result or a positive sputum culture for *M. tuberculosis*. Exclusion criteria included HIV infection, autoimmune or autoinflammatory diseases, chronic inflammatory disorders, current immunosuppressive therapy, major metabolic or other severe comorbid conditions, significant nutritional disorders, active smoking, and non-TB pulmonary diseases that could substantially confound the diagnosis or interpretation of pulmonary TB. Other clinical conditions considered likely to markedly influence systemic immune status or CD161-related measurements were also excluded based on the available clinical information at enrollment.

TB severity was classified using predefined, study-specific criteria developed with reference to previously reported approaches for assessing pulmonary TB disease severity and adapted according to the clinical and radiographic characteristics of our cohort. Previous studies and clinical guidance have identified the extent of pulmonary involvement, cavitation, and structural parenchymal damage as important indicators of disease burden in pulmonary TB. In particular, the WHO definition of extensive/advanced pulmonary TB incorporates bilateral cavitary disease or extensive parenchymal damage on chest radiography (WHO Guidelines Approved by the Guidelines Review Committee, 2024b), while the chest radiographic severity score proposed by Ralph et al. evaluates disease severity based on the percentage of lung affected together with the presence of cavitation (Ralph et al., 2010). These previously established concepts were considered when developing the present classification; however, the specific criteria used in this study were adapted for practical application to the clinical and imaging characteristics of our patient cohort rather than directly reproducing either of these existing systems.

Mild TB was defined as lesions involving ≤2 lung lobes without lung destruction, cavitation, emphysema, bronchiectasis, bronchial stenosis, or pleural effusion. Severe TB was defined as lesions involving ≥3 lung lobes, or lesions involving ≤2 lung lobes with at least one of these radiographic abnormalities. To facilitate interpretation of the severity classification, representative chest CT images from patients classified as mild TB and severe TB were selected to illustrate the typical radiographic features of the two groups and are provided in **Supplementary Figure 1**. The severe TB case demonstrated a cavitary lesion with surrounding parenchymal consolidation and more extensive structural involvement, whereas the mild TB case showed relatively limited pulmonary abnormalities without obvious cavitation or extensive lung destruction. These representative images are illustrative only, whereas severity classification for each patient was based on the complete predefined criteria described above. This classification was designed to provide a practical stratification of disease severity within the present cohort and was not intended to represent a universally validated staging system for pulmonary TB.

### Flow cytometric analysis of CD161-positive cell frequencies

Peripheral blood samples were obtained from 27 healthy donors and 57 TB patients. A total of 100 μl of whole blood was incubated with 1 ml hemolysin for 30 min in the dark, centrifuged (2000 rpm, 5 min), washed with phosphate-buffered saline (PBS), and stained with fluorochrome-conjugated antibodies for 30 min in the dark. After washing and resuspension in PBS, samples were analyzed by flow cytometry (Beckman Coulter, Brea, CA, USA). Data were analyzed using FlowJo version 10.0. The gating strategy was based on lymphocyte light-scatter characteristics followed by selection of CD3^+^ T cells and CD3^+^CD4^+^, CD3^+^CD8^+^, and CD3^+^Vδ2^+^ subsets. The CD3^−^CD56^+^ lymphocyte population was identified in this dataset; CD16 was not included in the staining panel.

### ELISA detection of soluble CD161 in plasma

Plasma samples from 52 HCs, 6 CAP patients, 5 individuals with LTBI, and 63 TB patients were diluted 1:5 and applied to pre-coated ELISA plates. After incubation at 37 °C for 1 h, biotin-labeled anti-CD161 mAb 5G6 (0.5 μg/ml) and HRP-streptavidin were added sequentially. TMB substrate was used for color development, and absorbance at 450 nm was measured against a recombinant CD161 standard curve. Each sample was analyzed in duplicate, and the mean absorbance values were used for quantification. To account for inter-plate variation, a common reference standard and calibration curve were included on each plate, and sample concentrations were calculated relative to the corresponding plate-specific standard curve.

### Immunohistochemistry and immunofluorescence of lung and lymph node tissues

TB lesions were confirmed by hematoxylin and eosin (H&E) staining. Paraffin-embedded sections underwent antigen retrieval, peroxidase blocking, and BSA blocking, followed by incubation with primary antibodies against CD3, CD8, and CD161. HRP-conjugated secondary antibodies were applied, and visualization was performed using 3,3′-diaminobenzidine (DAB) with hematoxylin counterstaining. For immunofluorescence, fluorescent secondary antibodies were used, and slides were examined by fluorescence microscopy. For tissue immunohistochemical analysis, pulmonary tissue specimens from 5 patients with pulmonary TB and lymph node specimens from 5 patients with TB were examined. Non-lesional regions within the corresponding lung and lymph node tissue specimens from patients with TB were used as reference controls for comparison with TB-associated lesions.

Histopathological regions, including non-lesional, granulomatous, perigranulomatous, and necrotic areas, were identified based on the corresponding morphological features observed on H&E-stained sections. For each patient, five representative microscopic fields were selected for quantitative analysis. Cells showing distinct membranous CD161 staining that was clearly distinguishable from background staining were considered CD161-positive, whereas cells without specific membranous staining were considered negative. The same criteria for CD161-positive staining were applied consistently across all tissue sections. Image assessment was performed manually by two experienced clinical pathologists who were blinded to the clinical information and independently evaluated the sections. CD3-, CD8-, and CD161-positive cells were evaluated in the selected fields. For each patient, measurements from the five fields were averaged to obtain a single patient-level value, and the patient was considered the statistical unit for subsequent analyses.

### Screening of blocking and competitive anti-CD161 antibodies

For blocking assays, plates were coated overnight with recombinant CD161-Fc protein (10 μg/ml). After incubation with mAbs, biotin-labeled LLT1-Fc protein was added, followed by HRP-conjugated anti-biotin antibody and TMB detection. In competitive assays, mAbs were co-incubated with biotin-labeled LLT1-Fc protein. Absorbance was measured at 450 nm to assess blocking efficacy. Based on the initial screening, antibodies 10A3 and 13A1 were selected as representative blocking clones for the downstream functional experiments because both showed clear interference with recombinant CD161-Fc/LLT1-Fc binding. The present assay evaluates recombinant protein-protein interaction and does not by itself establish blockade of native cell-surface CD161–LLT1 binding.

### Functional assessment of CD161 in an ESAT6-CFP10 antigen-stimulated PBMC system

PBMCs were isolated from a healthy donor and activated with anti-CD3 and IL-2 for 24 h, followed by stimulation with recombinant ESAT6-CFP10 fusion protein (10 μg/ml) for an additional 24 h. To investigate the potential role of CD161 signaling during antigen stimulation, anti-CD161 mAb 10A3 or 13A1 (10 μg/ml), or the corresponding isotype-control IgG, was added as indicated. Culture supernatants were collected, and the concentrations of IL-4, IL-10, IL-6, IL-17A, IFN-γ, and TNF were measured using a human cytometric bead array (CBA) kit (BD Biosciences). This *in-vitro* PBMC stimulation system was used to assess the effects of CD161 blockade under ESAT6-CFP10 antigen stimulation and was not intended to model active Mycobacterium tuberculosis infection.

### Data analysis

Flow cytometry data were analyzed using FlowJo version 10.0, and statistical analyses were performed using GraphPad Prism 9.0. Quantitative data are expressed as mean ± standard deviation (SD) unless otherwise specified. Comparisons between two independent groups were performed using Student’s *t*-test for approximately normally distributed data or the Mann–Whitney *U* test for non-normally distributed data, as appropriate. Correlations between plasma soluble CD161 (sCD161) levels and the frequencies of membrane CD161-expressing lymphocyte populations were assessed using Pearson’s correlation analysis. All statistical tests were two-sided, and a *P* value < 0.05 was considered statistically significant.

## Results

### Characterization of CD161 mAbs and establishment of a customized sCD161 ELISA

Through three rounds of subcloning, 14 IgG-type anti-human CD161 mAb clones were generated (**Figure 1A**). Five antibodies detected CD161 on CD161/L929 cells and human peripheral blood cells by flow cytometry (**Figure 1B**), and three recognized CD161 in immunohistochemical assays using CD161/L929 cells and human tonsil tissue (**Figures 1C, D**). Two antibodies, 14G12 and 5G6, showed non-identical competition patterns and were selected as capture and detection reagents for the customized sCD161 ELISA (**Figures 2A, B**). The assay showed specific reactivity to recombinant CD161 and no detectable cross-reactivity to sCD4, sCD8, or sCD25 under the tested conditions (**Figure 2C**). The calibration range was 0.0156–1 ng/ml with R^2^ = 0.9996 (**Figure 2C**). Precision and stability results are provided in **Supplementary Tables 2**, **3**. Formal affinity determination and epitope mapping were not performed in this study.

**Figure 1:**
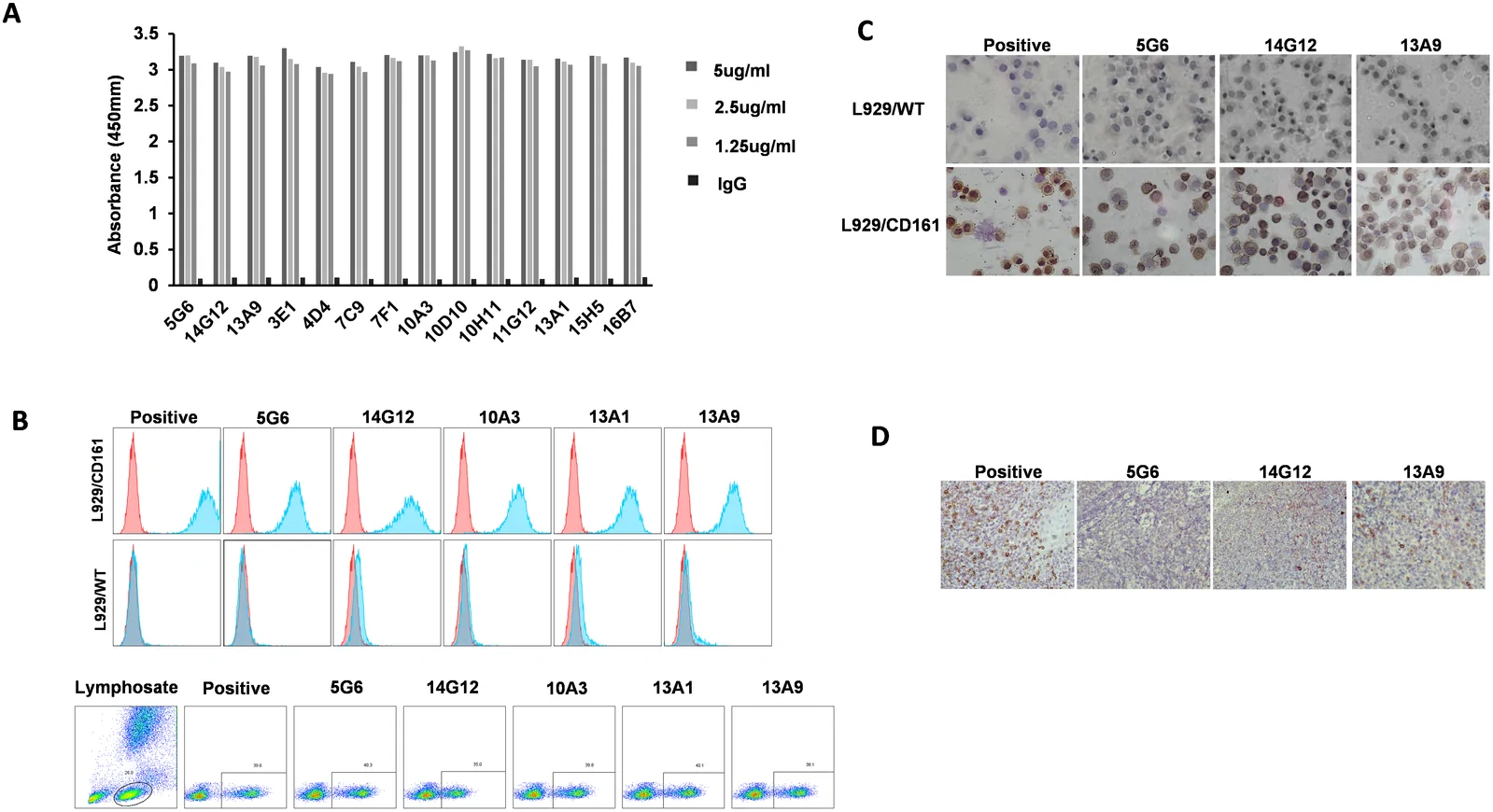
Characterization of newly generated anti-human CD161 monoclonal antibodies. **(A)** ELISA assessment of antibody binding to immobilized recombinant CD161-His. Data are presented as mean ± SD from three independent experiments. **(B)** Flow cytometry assessment of antibody reactivity with CD161-expressing lymphocytes in human peripheral blood. **(C)** Screening of antibodies for reactivity with CD161-expressing L929 cells. **(D)** Validation of antibody reactivity in fixed human tonsil tissue.

**Figure 2:**
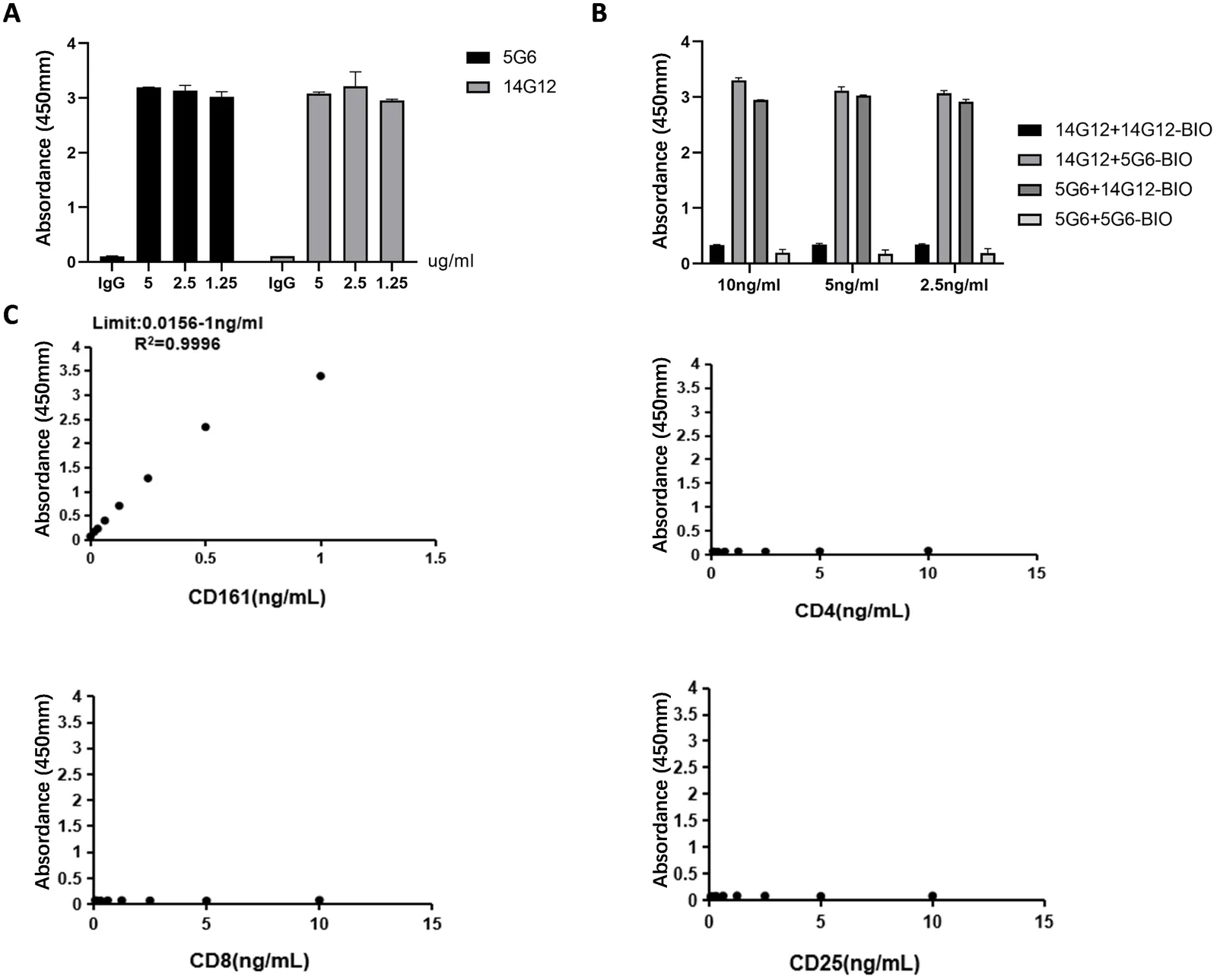
Establishment and analytical characterization of an in-house sandwich ELISA for soluble CD161 (sCD161). **(A)** Competition assay showing distinct competition patterns between mAbs 5G6 and 14G12. **(B)** Optimization of capture and detection antibody concentrations, with 14G12 selected as the capture antibody and biotinylated 5G6 as the detection antibody. **(C)** Specificity and calibration curve of the sCD161 ELISA using recombinant CD161. The calibration range was 0.0156–1 ng/mL, with R² = 0.9996. Assay specificity was evaluated using recombinant sCD4, sCD8, and sCD25.

### Plasma sCD161 is associated with TB and radiographic disease severity

Plasma sCD161 was measured in 52 HCs, 6 CAP patients, 5 LTBI individuals, and 63 untreated TB patients (**Figures 3A, B**). TB patients were classified as mild (n=20) or severe (n=43) according to the radiographic criteria described in Methods. Compared with HCs, plasma sCD161 was higher in TB patients (109.3 ± 32.40 vs. 135.2 ± 50.73, P = 0.0131). Severe TB patients had higher sCD161 levels than HCs (109.3 ± 32.40 vs. 144.7 ± 53.54, P = 0.0019) and CAP patients (100.6 ± 13.12 vs. 144.7 ± 53.54, P = 0.0202), whereas the difference between mild TB and HCs was not significant. Severe TB patients also had higher sCD161 levels than mild TB patients (115.0 ± 37.79 vs. 144.7 ± 53.54, P = 0.0299). Subgroup analyses showed higher sCD161 in TB patients with cavitary lesions, other pulmonary imaging abnormalities, extrapulmonary TB, or coinfection with other pulmonary pathogens compared with HCs (**Figure 3C**). These data support an association between circulating sCD161 and clinical/radiographic features of TB in this cohort but do not establish diagnostic accuracy or prognostic value.

**Figure 3:**
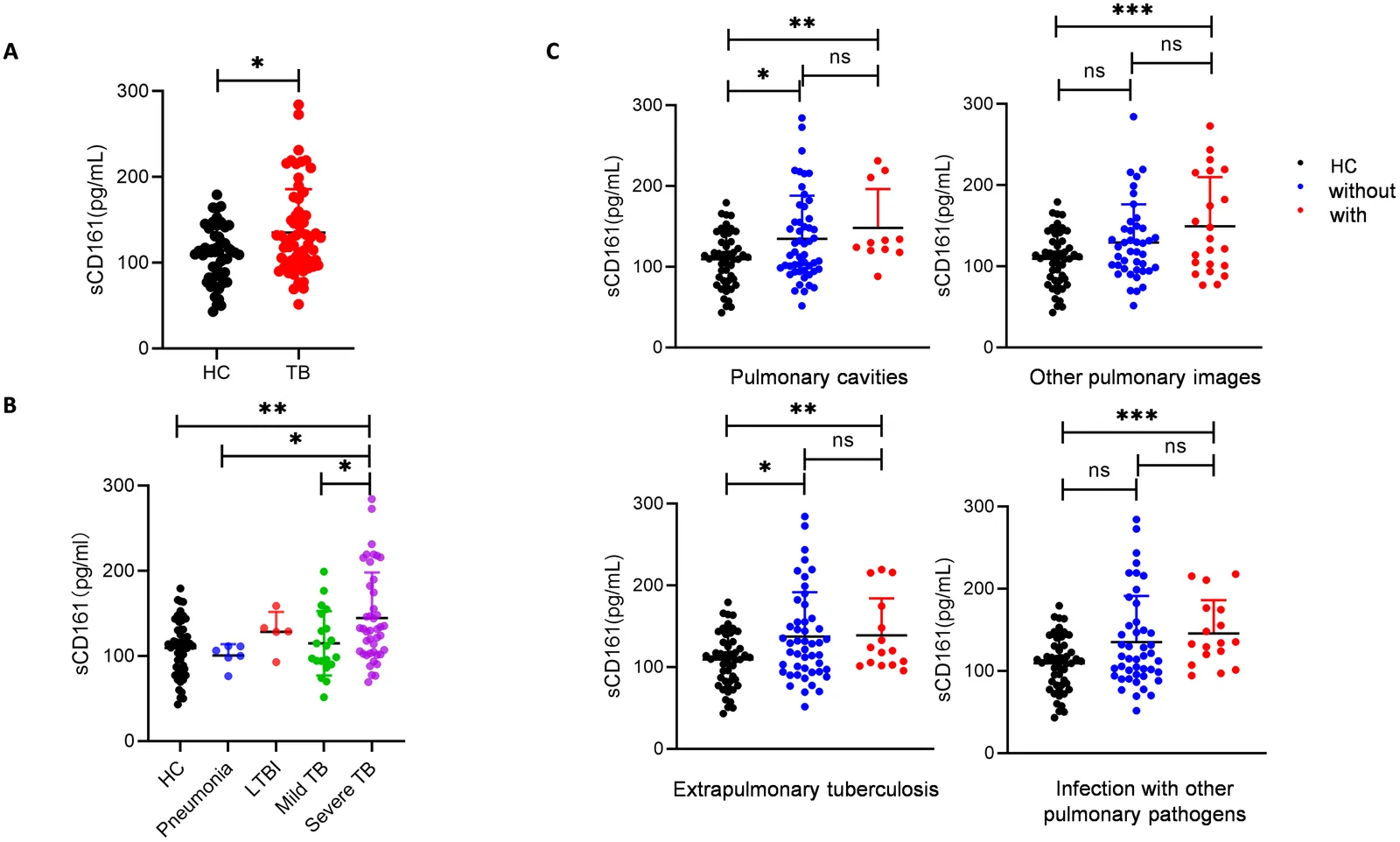
Plasma soluble CD161 levels in the study cohorts. **(A)** Plasma soluble CD161 (sCD161) levels in healthy controls (HC, n = 52) and patients with tuberculosis (TB, n = 63). **(B)** Plasma sCD161 levels in HC (n = 52), patients with community-acquired pneumonia (CAP, n = 6), individuals with latent tuberculosis infection (LTBI, n = 5), and patients with mild TB (n = 20) or severe TB (n = 43). **(C)** Plasma sCD161 levels in patients with TB stratified according to selected clinical and radiographic features. Individual data points are shown. ns: not significant, *P<0.05, **P<0.01, ***P<0.001.

### CD161-positive T-cell frequencies are reduced in peripheral blood of TB patients

Using mAb 5G6, we analyzed peripheral blood from 57 TB patients and 27 HCs (**Figures 4A–C**). TB patients had lower frequencies of CD161^+^/CD3^+^ T cells, CD161^+^/CD3^+^CD4^+^ T cells, and CD161^+^/CD3^+^Vδ2^+^ T cells than HCs (21.3% vs. 13.8%, P = 0.0005; 13.4% vs. 10.4%, P = 0.004; 69.97% vs. 60.03%, P = 0.038, respectively) (**Figure 4B**). Severe TB was associated with lower CD161^+^/CD3^+^ and CD161^+^/CD3^+^CD4^+^ T-cell frequencies, whereas mild TB showed a reduction in CD161^+^/CD3^+^CD4^+^ T cells (**Figure 4C**). Subgroup analyses according to pulmonary cavities, other pulmonary imaging abnormalities, extrapulmonary tuberculosis, and infection with other pulmonary pathogens are shown in **Figures 4D–G**. These measurements represent the frequency of CD161-positive cells within each population; they should not be interpreted as direct evidence of reduced receptor abundance per cell unless CD161 fluorescence intensity and/or absolute counts are also analyzed.

**Figure 4:**
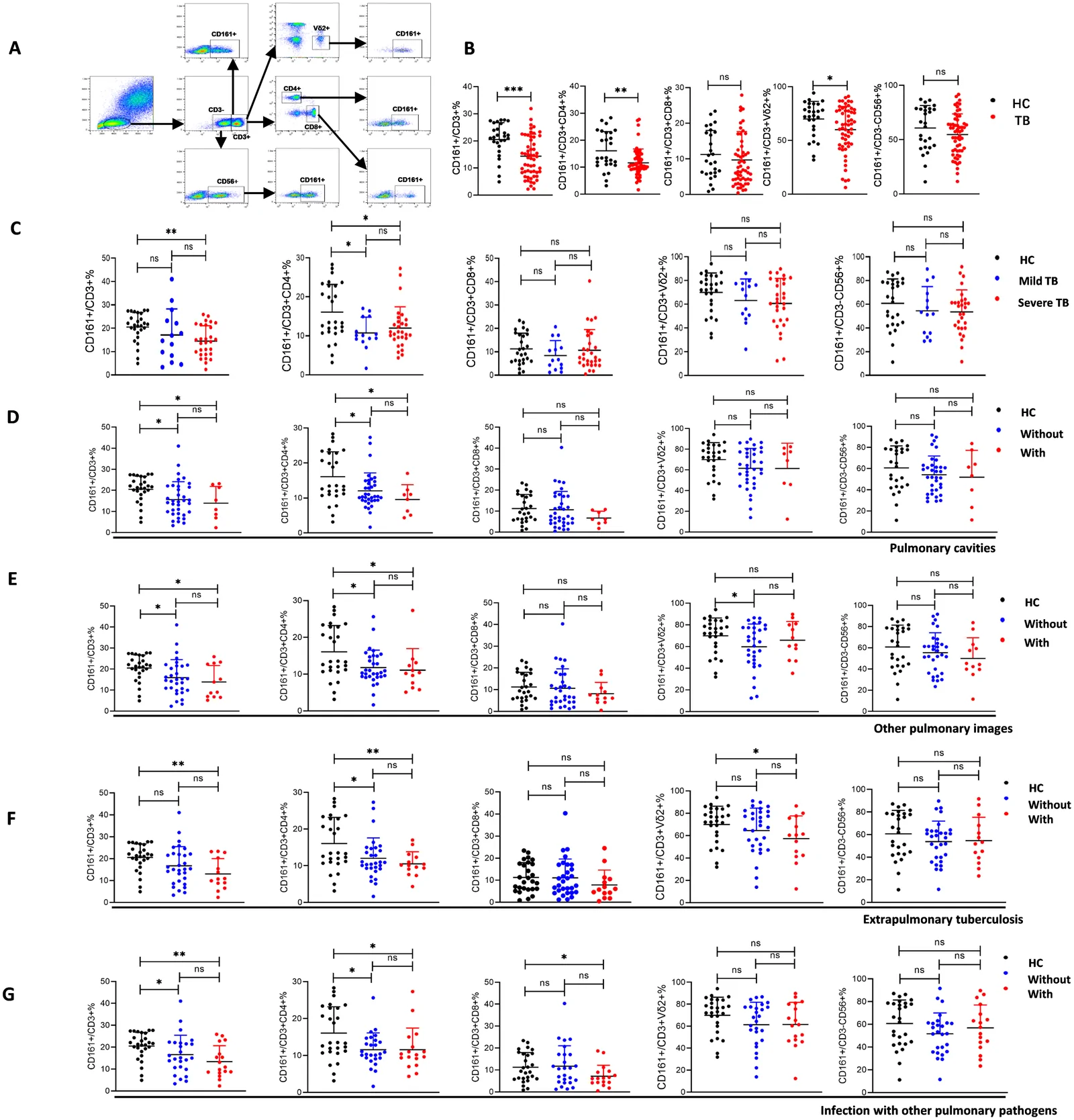
Frequencies of CD161-positive cell populations in peripheral blood. **(A)** Flow cytometry gating strategy for the identification of lymphocytes, CD3⁺ T cells, CD4⁺ T cells, CD8⁺ T cells, Vδ2⁺ T cells, and CD3⁻CD56⁺ lymphocytes. **(B)** Comparison of CD161-positive cell frequencies between healthy controls (HC, n = 27) and patients with tuberculosis (TB, n = 57) within the indicated cell populations. **(C)** Comparison of CD161-positive cell frequencies among HC (n = 27), mild TB (n = 14), and severe TB (n = 29) groups. **(D–G)** CD161-positive cell frequencies in patients with TB stratified according to pulmonary cavities, other pulmonary imaging abnormalities, extrapulmonary tuberculosis, and infection with other pulmonary pathogens, respectively. ns: not significant, *P<0.05, **P<0.01, ***P<0.001.

### Plasma sCD161 shows weak inverse associations with selected membrane CD161-positive T-cell frequencies

Plasma sCD161 showed weak inverse associations with the frequencies of CD161^+^/CD3^+^ T cells and CD161^+^/CD3^+^CD8^+^ T cells (r=−0.26, P = 0.02; r=−0.22, P = 0.04, respectively) (**Figures 5A, B**). Associations with CD161^+^/Vδ2^+^ T cells and CD161^+^/CD56^+^ cells were negative but did not reach statistical significance (r=−0.20, P = 0.08; r=−0.19, P = 0.10), and no association was observed with CD161^+^/CD3^+^CD4^+^ T cells (r=−0.02, P = 0.85). Given the small effect sizes and cross-sectional design, these findings should be interpreted as weak associations rather than evidence of a strong biological inverse relationship or membrane-to-soluble precursor-product relationship.

**Figure 5:**
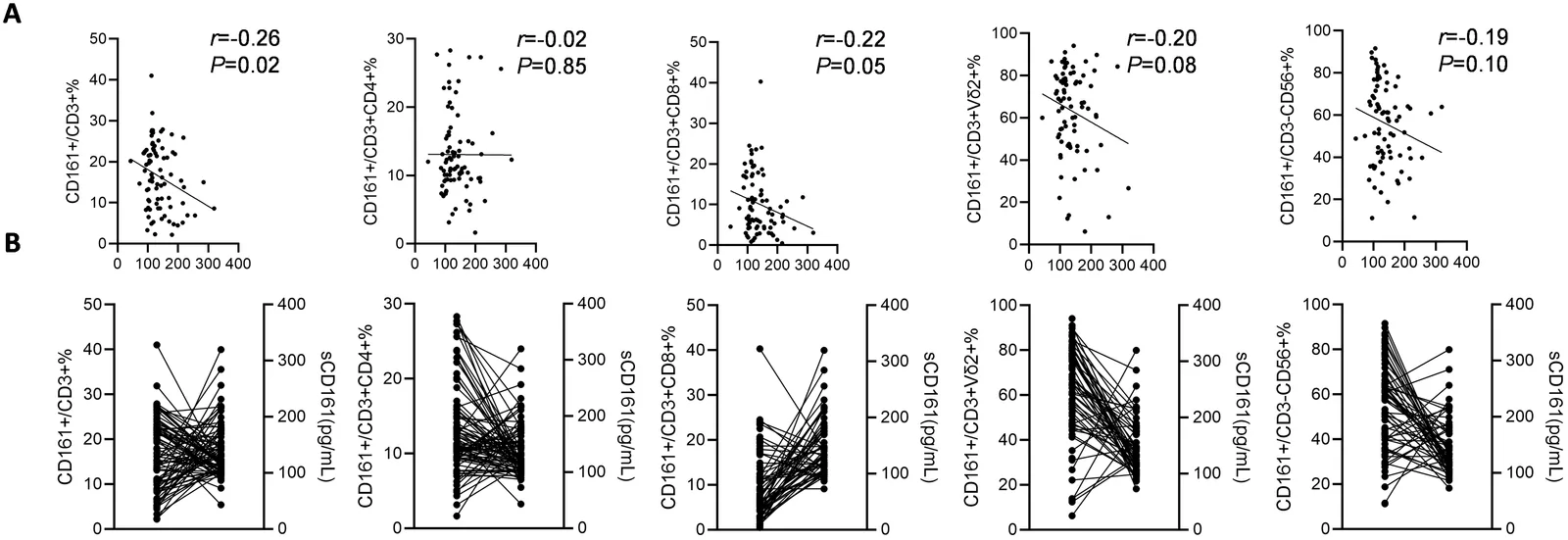
Associations between circulating sCD161 levels and membrane CD161-positive cell frequencies. **(A)** Scatterplots showing the associations between plasma sCD161 concentrations and the frequencies of membrane CD161-positive cell populations in participants with paired measurements, including 27 healthy controls and 57 patients with TB. Correlations were assessed using two-tailed Pearson’s correlation analysis. Pearson’s correlation coefficients (r) and P values are shown for each analysis. **(B)** Paired visualization of plasma sCD161 concentrations and the corresponding frequencies of membrane CD161-positive cell populations in the same participants. Lines connect the plasma sCD161 measurement and the corresponding membrane CD161-positive cell frequency from the same individual.

### Spatial distribution of CD161 in TB lung and lymph-node lesions

H&E staining confirmed necrotic areas and granulomatous inflammation in TB tissues (**Figure 6A**). CD161 immunohistochemistry showed higher staining in perigranulomatous lung tissue than in non-lesional reference lung tissue and lower staining in necrotic than in granulomatous regions (**Figures 6B–D**). Similar spatial patterns were observed in lymph nodes (**Figure 6E**). Immunofluorescence showed increased proportions of CD3^+^CD161^+^ and CD8^+^CD161^+^ T cells in perigranulomatous lung and lymph-node tissues compared with non-lesional reference tissues (**Figures 7A–F**). These findings indicate a spatial association of CD161-positive cells with granulomatous tissue.

**Figure 6:**
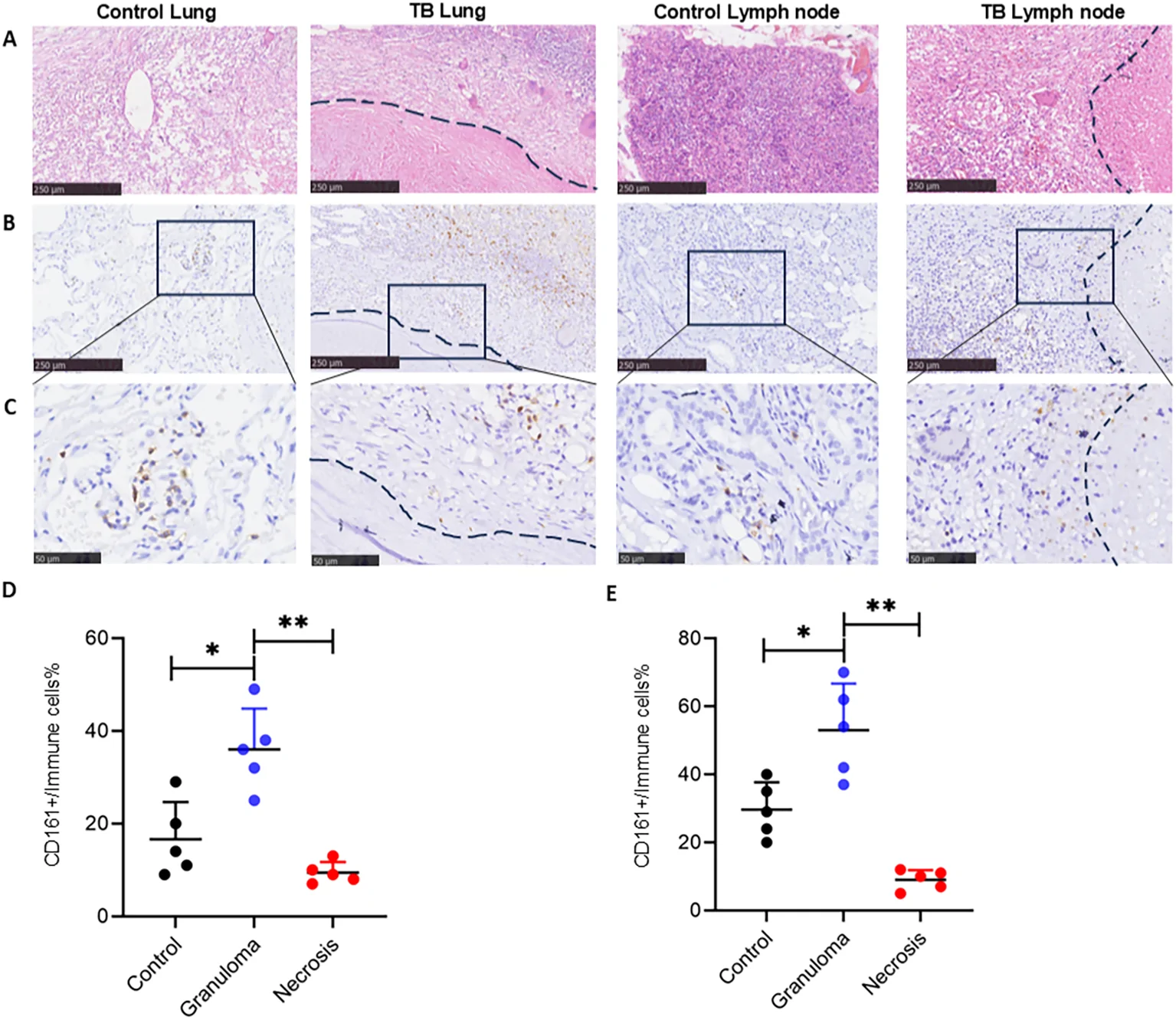
Spatial distribution and quantification of CD161 in TB lung and lymph-node lesions. **(A)** Representative H&E staining. **(B)** CD161 immunohistochemical staining in lung and lymph-node tissues at 100× magnification. **(C)** CD161 immunohistochemical staining at 400× magnification. **(D–E)** Quantification of CD161-positive cells in control, granulomatous, and necrotic regions of lung and lymph-node tissues, respectively. Pulmonary tissue specimens from 5 patients with pulmonary TB and lymph-node specimens from 5 patients with TB were analyzed. Non-lesional regions within the corresponding tissue specimens from patients with TB were used as reference controls. Five representative microscopic fields were analyzed for each patient, and measurements were averaged to obtain a single patient-level value. *P<0.05, **P<0.01.

**Figure 7:**
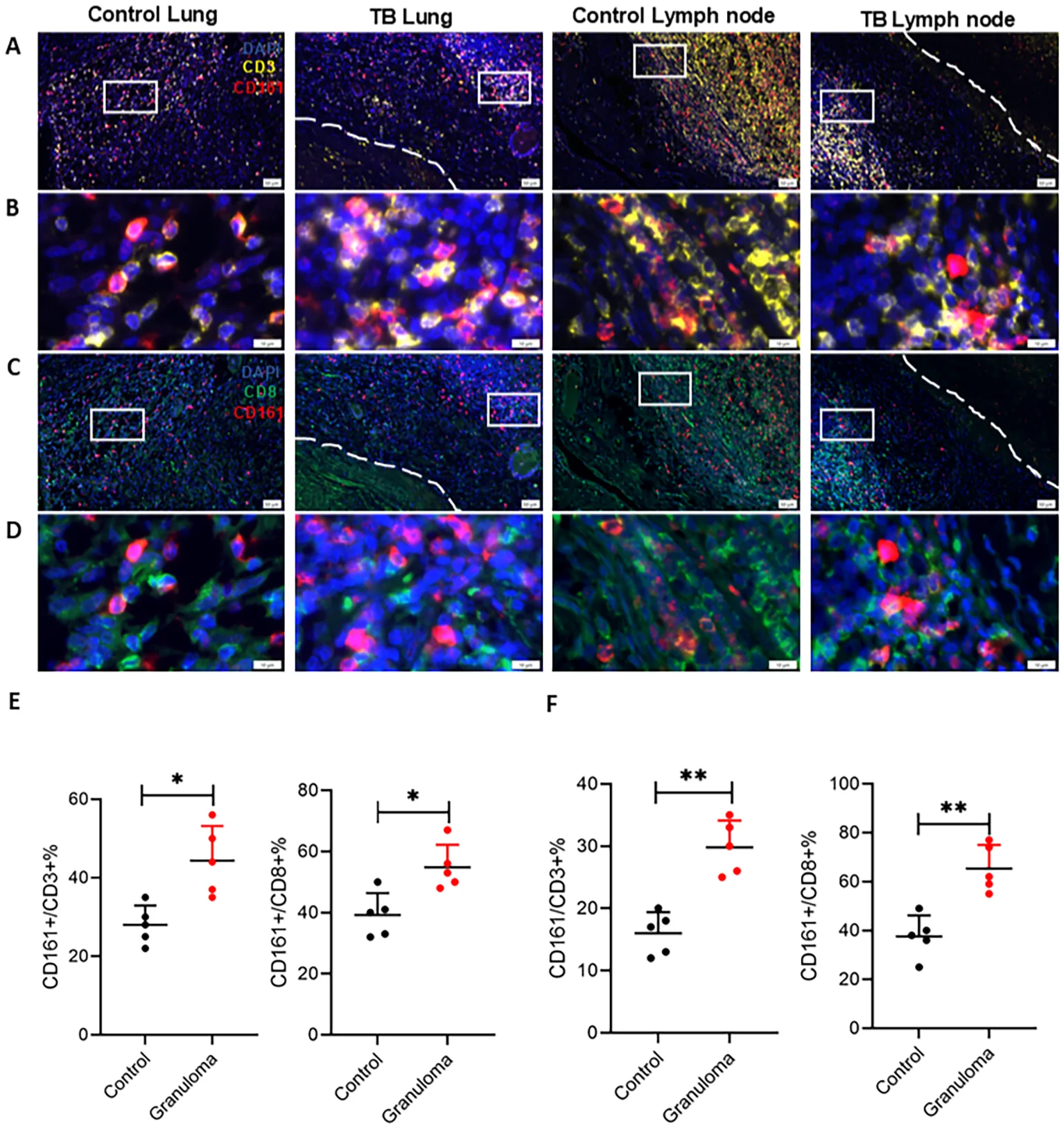
CD3, CD8, and CD161 co-expression in TB lesions. **(A–D)** Representative co-staining of CD3/CD161 and CD8/CD161 in control and TB lesion regions of lung and lymph-node tissues. **(E–F)** Quantification of CD3⁺CD161⁺/CD3⁺ and CD8⁺CD161⁺/CD8⁺ proportions in the indicated tissue regions. Pulmonary tissue specimens from 5 patients with pulmonary TB and lymph-node specimens from 5 patients with TB were analyzed. Non-lesional regions within the corresponding tissue specimens from patients with TB were used as reference controls. Five representative microscopic fields were analyzed for each patient, and measurements were averaged to obtain a single patient-level value. Data are presented as mean ± SD. *P<0.05, **P<0.01.

### Identification of CD161-blocking antibodies

Several mAbs inhibited recombinant CD161-Fc/LLT1-Fc binding in ELISA-based assays (**Figures 8A, B**). Competitive assays identified clones 13A1, 13A9, and 10A3 as blocking antibodies, and inhibition was dose dependent (**Figures 8C–E**). Clones 10A3 and 13A1 were selected as representative blocking antibodies for the downstream cellular experiments.

**Figure 8:**
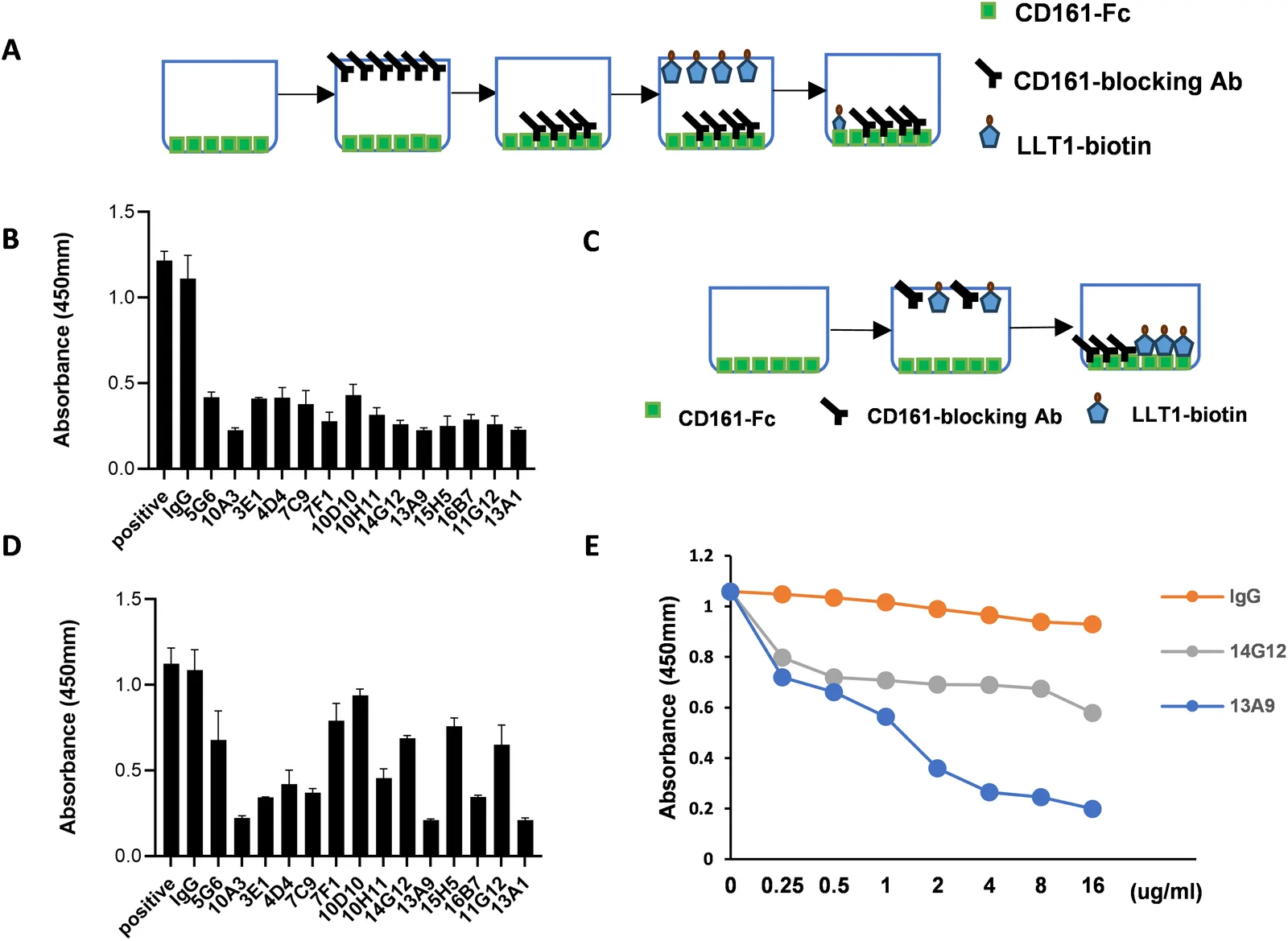
Screening of blocking anti-CD161 antibodies. **(A)** Experimental workflow. **(B)** Identification of mAbs that inhibit recombinant CD161-Fc/LLT1-Fc binding. **(C)** Competitive blocking assay. **(D)** Representative clones showing competition with LLT1. **(E)** Dose-dependent inhibition. Data are presented as mean ± SD from three independent experiments.

### CD161–LLT1 blockade alters cytokine responses in an ESAT6-CFP10 antigen-stimulated PBMC system

ESAT6-CFP10 stimulation increased IL-6, IL-17A, IFN-γ, and TNF secretion, whereas IL-4 and IL-10 were not significantly altered under the tested conditions (**Figures 9A–F**). In the presence of blocking antibodies, clone 10A3 reduced IL-6 and TNF while increasing IL-17A, whereas clone 13A1 reduced IL-6, IFN-γ, and TNF while increasing IL-17A. Because cytokines were measured in bulk PBMC supernatants, these findings demonstrate altered cytokine responses after CD161–LLT1 blockade but do not establish direct regulation of Th1 or Th17 cells or identify the cellular source of each cytokine.

**Figure 9:**
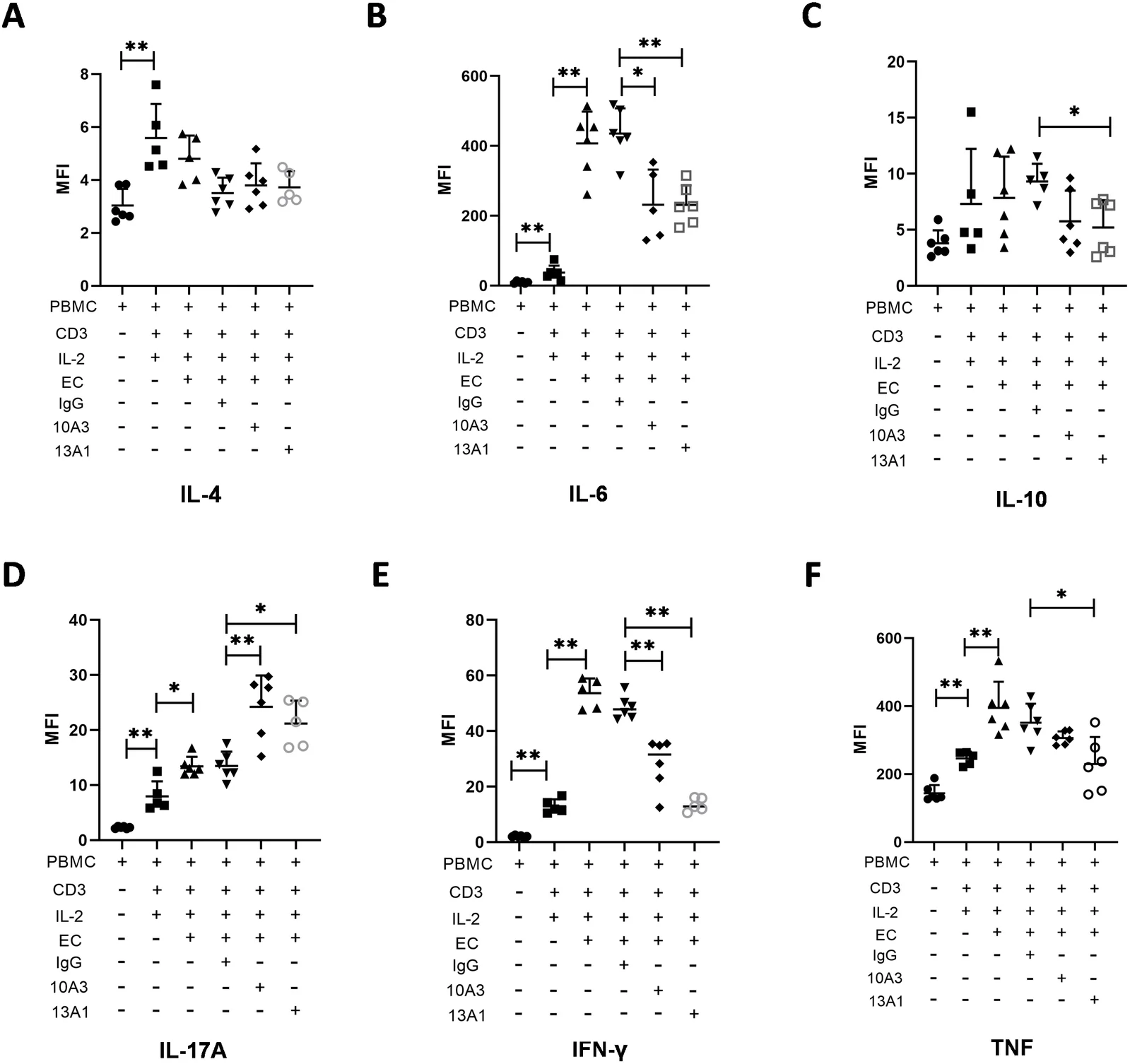
Cytokine production in PBMCs following CD161–LLT1 blockade in an ESAT6-CFP10 antigen-stimulated PBMC system. PBMCs from one healthy donor were stimulated with ESAT6-CFP10 in the presence of a CD161-blocking antibody (10 μg/mL) or an isotype-matched IgG control. **(A)** IL-4, **(B)** IL-6, **(C)** IL-10, **(D)** IL-17A, **(E)** IFN-γ, and **(F)** TNF. Three independent experiments were performed, with each condition tested in duplicate wells. Data are presented as mean ± SD.

In this single-center cross-sectional cohort, plasma sCD161 was elevated in patients with active pulmonary TB, particularly in those classified as severe according to radiographic criteria. We also observed lower frequencies of CD161-positive T-cell subsets in peripheral blood and distinct spatial patterns of CD161 staining in TB-associated tissues. In an ESAT6-CFP10 antigen-stimulated PBMC system, blocking CD161–LLT1 altered the cytokine profile. Together, these observations support an association between CD161 dysregulation and TB-associated immune responses, while the mechanistic and diagnostic implications require further validation.

## Discussion

The reduction in CD161^+^/CD3^+^CD4^+^ and CD161^+^/CD3^+^Vδ2^+^ T-cell frequencies in TB is consistent with previous reports of altered CD161-positive T-cell compartments during active disease (Chua et al., 2012; Yang et al., 2015; Provine et al., 2018; Yang et al., 2021). CD161-positive T cells can display rapid cytokine and cytotoxic responses to mycobacterial stimulation, but our cross-sectional data cannot determine whether reduced CD161-positive frequencies result from altered cell composition, receptor regulation, disease-related redistribution, or other processes. In particular, the present study did not directly measure CD161 surface intensity or absolute cell counts; therefore, reduced frequencies should not be interpreted as definitive receptor downregulation at the single-cell level.

The present study did not demonstrate a significant difference in the frequency of CD161^+^/CD3^+^CD8^+^ T cells between HC and TB groups. CD8^+^CD161^+^ T cells are heterogeneous and can exhibit cytotoxic, memory, and tissue-homing properties (Konduri et al., 2020). Their contribution to antimycobacterial immunity may therefore depend on differentiation and tissue context. Accordingly, our data do not support the conclusion that CD161 on CD4^+^ T cells is more critical than CD161 on CD8^+^ T cells; rather, they identify subset-specific differences that warrant mechanistic investigation.

The increase in circulating sCD161 and its weak inverse association with selected membrane CD161-positive T-cell frequencies raise the possibility that membrane-associated and soluble CD161 are biologically linked. However, the present study does not establish the origin of the plasma analyte or directly demonstrate a proteolytic shedding mechanism. Soluble immune checkpoints have been described in TB, and several, including sPD-1, sPD-L1, sLAG-3, and related molecules, can vary with disease status or treatment (Chen et al., 2022). Other soluble checkpoint proteins such as sCD137 have also been associated with TB severity and clinical outcomes in larger cohorts (Yi et al., 2024). Notably, a recent study in primary biliary cholangitis provided experimental evidence supporting a potential proteolytic shedding mechanism for sCD161. In that study, CD161 mRNA expression was not significantly reduced, whereas pharmacological inhibition of ADAM10/ADAM17 with GW280264X decreased sCD161 release into the culture supernatant and increased cell-surface CD161 expression on several immune-cell subsets (Liu et al., 2026). These findings support the possibility that ADAM10/ADAM17-mediated proteolytic shedding contributes to sCD161 release and may provide a biologically plausible explanation for the inverse relationship observed between circulating sCD161 and membrane CD161 expression in our cohort.

CD161 staining was enriched in perigranulomatous tissue and reduced in necrotic regions. CD161-positive populations, including tissue-homing and innate-like T-cell subsets, have been described in mucosal and inflammatory sites (Billerbeck et al., 2010; Gold et al., 2010; Dusseaux et al., 2011; Ussher et al., 2014; Ogongo et al., 2020; Balfour et al., 2021; Sakai et al., 2021; McInerney et al., 2024), and chemokine networks contribute to recruitment and organization of immune cells during TB (Lyu J. et al., 2024; Yin et al., 2026). However, the present tissue data provide descriptive spatial associations rather than proof that CD161-positive cells are specifically recruited by a defined chemokine program or that CD161 itself controls granuloma organization. Standardized region definitions and patient-level spatial quantification will be important to test these hypotheses.

The functional experiments should be interpreted within the limits of the *in-vitro* model. ESAT6-CFP10 is an Mtb antigenic stimulus and does not reproduce live Mtb infection, the granuloma microenvironment, or the full diversity of Mtb antigens and host-cell interactions. Therefore, our findings show that CD161–LLT1 blockade changes cytokine secretion in activated PBMCs exposed to ESAT6-CFP10, but they do not establish direct regulation of Th1 or Th17 cells. This distinction is important because bulk PBMC cultures contain multiple CD161-expressing T-cell subsets and NK cells. Recent work on other immune checkpoints, including TIGIT blockade in TB, illustrates the value of subset-specific and *in-vivo* validation when drawing mechanistic conclusions about checkpoint pathways (Zhou et al., 2025).

Overall, our findings indicate that circulating sCD161 is associated with active TB and radiographic disease severity in this cohort, while CD161–LLT1 blockade modulates cytokine responses in an antigen-stimulated PBMC system. Several limitations remain: the CAP and LTBI groups are small; there is no independent validation cohort or longitudinal follow-up; the severity definition is based primarily on radiographic criteria; the cellular source and molecular identity of plasma sCD161 were not directly established; and functional assays were performed in bulk PBMCs without subset-specific cytokine measurements. In addition, the current study does not demonstrate treatment efficacy or therapeutic benefit from CD161/LLT1 blockade. Future multicenter longitudinal studies with larger disease-control cohorts, validated analytical assays, direct molecular characterization of sCD161, and subset-specific functional experiments will be required to establish the clinical utility and mechanism of this pathway in TB.

## Data Availability

The raw data supporting the conclusions of this article will be made available by the authors, without undue reservation.

## Supplementary material

Supplementary Figure 1 Representative chest CT images of patients with different radiographic severity of pulmonary tuberculosis. **(A)** Representative CT images from a patient classified as severe TB, showing a cavitary lesion in the right upper lung accompanied by surrounding parenchymal consolidation and relatively extensive structural involvement. **(B)** Representative CT images from a patient classified as mild TB, showing relatively limited pulmonary parenchymal abnormalities without obvious cavitation or extensive structural lung destruction. These images are provided for illustrative purposes, and severity classification was based on the complete predefined radiographic criteria described in the Methods.
