# Supplementary material for: CD161 expression in tuberculosis and functional effects of CD161–LLT1 blockade
Source: Front Cell Infect Microbiol. 2026 Oct 28;16:1962964. doi: 10.3389/fcimb.2026.1962964 (PMC13751381; doi:10.3389/fcimb.2026.1962964)
Supplement: Supplementary file 1 [file Table1.docx]

**Supplement Table 1**

**Patient characteristics**

| **Characteristics** | **TB patients** | | **Healthy controls** | | **LTBI** | **Pneumonia** |
| --- | --- | --- | --- | --- | --- | --- |
| Age（Mean ± SD） | 42.47±18.36 | | | 40.67±10.12 | 45.40+15.88 | 45.67+12.91 |
| Sex | Men | 55（71.4%） | | 31（56.4%） | 1（20%） | 3（50%） |
|  | Women | 22（28.6%） | | 24（43.6%） | 4（80%） | 3（50%） |
| Severity of the disease | Mild TB | 20（26.0%） | | - | - | - |
|  | Severe TB | 43（55.8%） | | - | - | - |
| Combined with pulmonary cavities | Yes | 11（14.3%） | | - | - | - |
|  | No | 52（67.5%） | | - | - | - |
| Extrapulmonary tuberculosis | Yes | 15（19.5%） | | - | - | - |
|  | No | 48（62.3%） | | - | - | - |
| Combined with other pulmonary images | Yes | 22（28.6%） | | - | - | - |
|  | No | 41（53.2%） | | - | - | - |
| Infection with other pulmonary pathogens | Yes | 17（22.1%） | | - | - | - |
|  | No | 45（58.4%） | | - | - | - |

TB severity classification was available for 63 of 77 patients; 14 patients could not be classified because of incomplete clinical and radiographic information. Percentages are based on the total TB cohort (n = 77).

**Supplement Table 2**

**Precision of the human sCD161 ELISA system**

| Sample | Intra-assay precision | | | Inter-assay precision | | |
| --- | --- | --- | --- | --- | --- | --- |
|  | 1 | 2 | 3 | 1 | 2 | 3 |
| n | 10 | 10 | 10 | 10 | 10 | 10 |
| ‾x | 2.35 | 1.73 | 0.88 | 2.41 | 1.38 | 0.73 |
| SD | 0.016 | 0.011 | 0.007 | 0.20 | 0.02 | 0.07 |
| CV% | 3 | 3.7 | 5.4 | 8.1 | 1.7 | 9.6 |

**Supplement Table 3**

**Stability of the human sCD161 ELISA system.**

| Time | 0.5ng/ml | | 0.25ng/ml | | 0.125ng/ml | |
| --- | --- | --- | --- | --- | --- | --- |
|  | ‾x±s | CV% | ‾x±s | CV% | ‾x±s | CV% |
| 0 | 2.58±0.04 | 1.5 | 1.56±0.09 | 5.8 | 0.82±0.04 | 5.2 |
| 30 | 2.47±0.02 | 0.7 | 1.35±0.04 | 2.8 | 0.72±0.04 | 5.3 |
| 90 | 1.98±0.12 | 6.1 | 1.10±0.09 | 7.9 | 0.58±0.05 | 8.9 |
